# Decoding T cell exhaustion in the tumour microenvironment

**DOI:** 10.1038/s12276-026-01809-w

**Published:** 2026-08-31

**Authors:** Jin A Park, Jeyeon Im, Sung-Min Hwang

**Affiliations:** Department of Biological Sciences, Ulsan National Institute of Science and Technology (UNIST), Ulsan, 44919 Republic of Korea

**Keywords:** Tumour immunology, Immunotherapy

## Abstract

T cell exhaustion arises during chronic antigen stimulation and represents a major barrier to effective anti-tumour immunity. Rather than a uniform dysfunctional state, exhaustion is increasingly understood as a structured differentiation landscape that progresses from stem-like progenitor exhausted T cells to terminally exhausted T cells. Stem-like progenitor exhausted T cells retain self-renewal capacity and partial effector function, whereas terminally exhausted T cells exhibit epigenetically fixed dysfunction and limited cytokine production. Within the tumour microenvironment, persistent antigen stimulation, together with metabolic stressors such as hypoxia and nutrient deprivation, accelerates this differentiation trajectory, thereby constraining protective immunity. In this review, we conceptualize T cell exhaustion as a dynamic continuum shaped by both differentiation states and microenvironmental niches. We outline an integrative framework to define exhaustion by combining antigen experience, cellular phenotype, functional capacity, epigenetic fixation and spatial context. Viewing immunotherapies through this multidimensional framework highlights the notion that durable therapeutic responses depend on preserving stem-like progenitor exhausted T cells within supportive niches and preventing their terminal differentiation. Understanding how these cellular states are maintained or disrupted within the tumour microenvironment will provide new opportunities for designing therapies that sustain protective T cell immunity in cancer.

## T Cell Exhaustion Defined by Differentiation and Microenvironments

T cell exhaustion in the tumor microenvironment is a significant barrier to effective cancer immunotherapy, often leading to reduced efficacy of immune checkpoint blockade therapies. This review explores the complex differentiation landscape of exhausted T cells, shaped by chronic antigen exposure and the spatial and metabolic constraints of the tumour microenvironment. The authors discuss how cancer-associated fibroblasts and tumour-associated macrophages create immunosuppressive niches that promote T cell exhaustion. They highlight the role of metabolic competition, such as amino acid and glucose depletion, in reinforcing exhaustion. Key findings include the identification of distinct exhausted T cell subsets, such as progenitor exhausted and terminally exhausted cells, and their spatial distribution within tumours. The review emphasizes the need for integrated therapeutic strategies, combining multi-checkpoint blockade and metabolic reprogramming, to overcome exhaustion and enhance immune checkpoint blockade efficacy.

This summary was initially drafted using artificial intelligence, then revised and fact-checked by the author.

## Introduction

T cell exhaustion within the tumour microenvironment (TME) represents a critical bottleneck in anti-tumour immunity and is a major determinant of responsiveness and durability of immune-checkpoint blockade (ICB) therapies^1,2^. Tumour-associated T cells were long viewed as converging towards a single terminal state characterized by high expression of inhibitory receptors and diminished effector function, a simplification that has obscured the biological complexity underlying therapeutic outcomes. Recent advances in single-cell and spatial profiling technologies now reveal that T cell exhaustion is organized as a structured differentiation landscape shaped by chronic antigen stimulation, TME niches and metabolic and immunosuppressive pressures that enforce both functional constraints and lineage stability^3^. These findings support the view that exhaustion is not merely a dysfunctional state but an adaptive differentiation program that progresses from progenitor to intermediate and ultimately to terminal states^1^.

Cytotoxic T cell exhaustion was initially described in the context of chronic lymphocytic choriomeningitis virus infection in mice, in which virus-specific effector CD8^+^ T cells appeared to be lost. Subsequent studies showed that these cells persist but adopt a distinct functional state, establishing exhaustion as a defined T cell fate rather than simple cellular attrition. Early adoptive transfer experiments further demonstrated that removal of exhausted T cells from antigen-rich environments does not restore normal memory T cell properties^2,4,5^, indicating that exhaustion reflects a stably imprinted program rather than a transient or reversible dysfunction.

In this Review, we synthesize recent advances in understanding the cellular and molecular architecture of T cell exhaustion with the TME and discuss how this framework may guide the development of next-generation immunotherapies.

## Mechanisms shaping T cell exhaustion in the tumour microenvironment

### Spatial organization

Within the TME, T cell exhaustion does not arise randomly but instead follows differentiation trajectories shaped by the spatial architecture of tumours. Stromal and myeloid compartments organize specialized microenvironments that regulate the localization, activation state and differentiation of tumour-infiltrating T cells^6^. Cancer-associated fibroblasts (CAFs) originate from tissue fibroblasts reprogrammed by tumour-derived signals and represent a dominant stromal compartment of the TME^7^. CAFs promote regulatory T cell (Treg) accumulation while suppressing cytotoxic immune-cell activity^8^. Mechanistically, CAF-derived TGF-β promotes Treg cell expansion, whereas CAFs further reduce IL-2 availability by limiting IL-2 production and expanding activated Tregs, thereby reinforcing an immunosuppressive microenvironment^9^. Furthermore, CAF-expressed inhibitory molecules, including PD-L2 and FASL, impair antigen-specific CD8^+^ T cell survival and effector function, further limiting cytotoxic anti-tumour immunity^10^. In addition, CAFs upregulate immune-checkpoint ligands such as PD-L1, PD-L2 and CD80/CD86, thereby engaging PD-1 and CTLA-4 on T cells and promoting exhaustion programs^11^. CAF-rich regions also remodel the extracellular matrix (ECM) through secretion of matrix-degrading enzymes and enhanced matrix deposition and crosslinking^8^. These changes increase ECM alignment, stiffness and density, generating fibrotic barriers that physically restrict T cell infiltration and limit interactions between T cells and tumour cells^12^. Consequently, CAF-rich stromal areas constitute prototypical immunosuppressive niches that promote exhaustion.

Tumour-associated macrophages (TAMs) represent another dominant immune population in most solid tumours, and their abundance often correlates with poor clinical outcomes^7^. Within the TME, stromal and myeloid cells interact to establish stromal–myeloid suppressive networks in which CAFs and TAMs engage in reciprocal signalling circuits^13^. Antigen-specific CD8^+^ T cells are not randomly distributed but instead preferentially accumulate in TAM-rich regions. Although TAMs can present antigen, they inefficiently support productive T cell activation and proliferation, instead delivering signals that reinforce exhaustion programs. Hypoxic regions frequently contain dense clusters of TAMs, generating microenvironments that further accelerate exhaustion^14^. Spatial analyses of tumours subdivided into outer rim, intermediate regions and inner core compartments reveal that naïve CD8^+^ T cells are enriched in peripheral tumour areas, whereas exhausted CD8^+^ T cells accumulate towards the tumour interior^14^. This spatial gradient is reinforced by feed-forward circuits in which CSF1 produced by exhausted T cells promotes TAM maturation, and mature TAMs in turn reinforce exhausted states by limiting effective antigen presentation^14^.

CAF–TAM crosstalk further stabilizes these suppressive niches. CAFs sense tissue density and regulate CSF1 expression via the Hippo–YAP signalling, thereby controlling macrophage abundance and spatial distribution via CSF1R signalling^7,15–17^. In addition, CAFs orchestrate monocyte recruitment and TAM differentiation through chemokine and cytokine pathways including the CXCL12–CXCR4 and CCL2–CCR2 axes^18–20^. Together, these stromal-myeloid circuits generate stable immunosuppressive niches that sustain CD8^+^ T cell dysfunction despite persistent antigen stimulation^7,13^.

Spatial profiling studies further reveal that tumours contain organized immune niches composed of CD8^+^ T cells, dendritic cells and MHCII^+^ macrophages localized around vascular structures. These perivascular immune niches create a localized inflammatory microenvironment that supports T cell activation and differentiation^21^. In multiple tumour models, progenitor exhausted T cells (T_PEX_, TCF1^+^PD-1^+^CD8^+^ T cells) preferentially reside within these niches, whereas terminally exhausted T cells (T_TEX_) accumulate within tumour parenchyma. For example, in the KP-NINJA lung-cancer model, TCF1^+^PD-1^+^CD8^+^ T cells are approximately 10-fold more abundant in tumour-draining lymph nodes than in tumours, and the size of this T_PEX_ pool remains relatively stable during tumour progression. By contrast, early KP lung tumours expressing neoantigens initially display an immunologically hot TME that progressively transitions into an immunologically cold TME characterized by T cell exclusion from the tumour parenchyma and confinement to tertiary lymphoid structures (TLS)^22^. Consistently, in head and neck squamous cell carcinoma, CD8^+^PD-1^+^TCF1^−^ T_TEX_ cells are more frequently detected within tumours, whereas TCF1^+^ T_PEX_ cells are significantly enriched in tumour-draining lymph nodes. Moreover, both T_PEX_ and T_TEX_ populations referentially localize to stromal regions surrounding tumours rather than the tumour parenchyma itself^23^. T_PEX_ cells (TCF1^+^PD-1^+^CD8^+^ T cells) are rarely observed inside tumour parenchyma but instead localize to TLSs within and around tumours, where they are maintained in a quiescent and protected niche^4^. Collectively, these observations indicate that exhausted T cell subsets are spatially biased toward TLSs, perivascular regions and immune-cell-rich stromal niches rather than the tumour core, and that such spatial organization is a central determinant of exhaustion differentiation trajectories.

### Metabolic constraints

Metabolic competition within the TME is considered a major mechanism that can progressively erode T cell fitness. Tumour cells, stromal cells and immunosuppressive myeloid populations reshape the metabolic landscape of tumours by consuming essential nutrients, generating inhibitory metabolites and imposing hypoxic stress. Together, these metabolic constraints can disrupt bioenergetic homeostasis in tumour-infiltrating T cells and reinforce exhaustion programs.

#### Amino acid metabolism

**Fig. 1:**
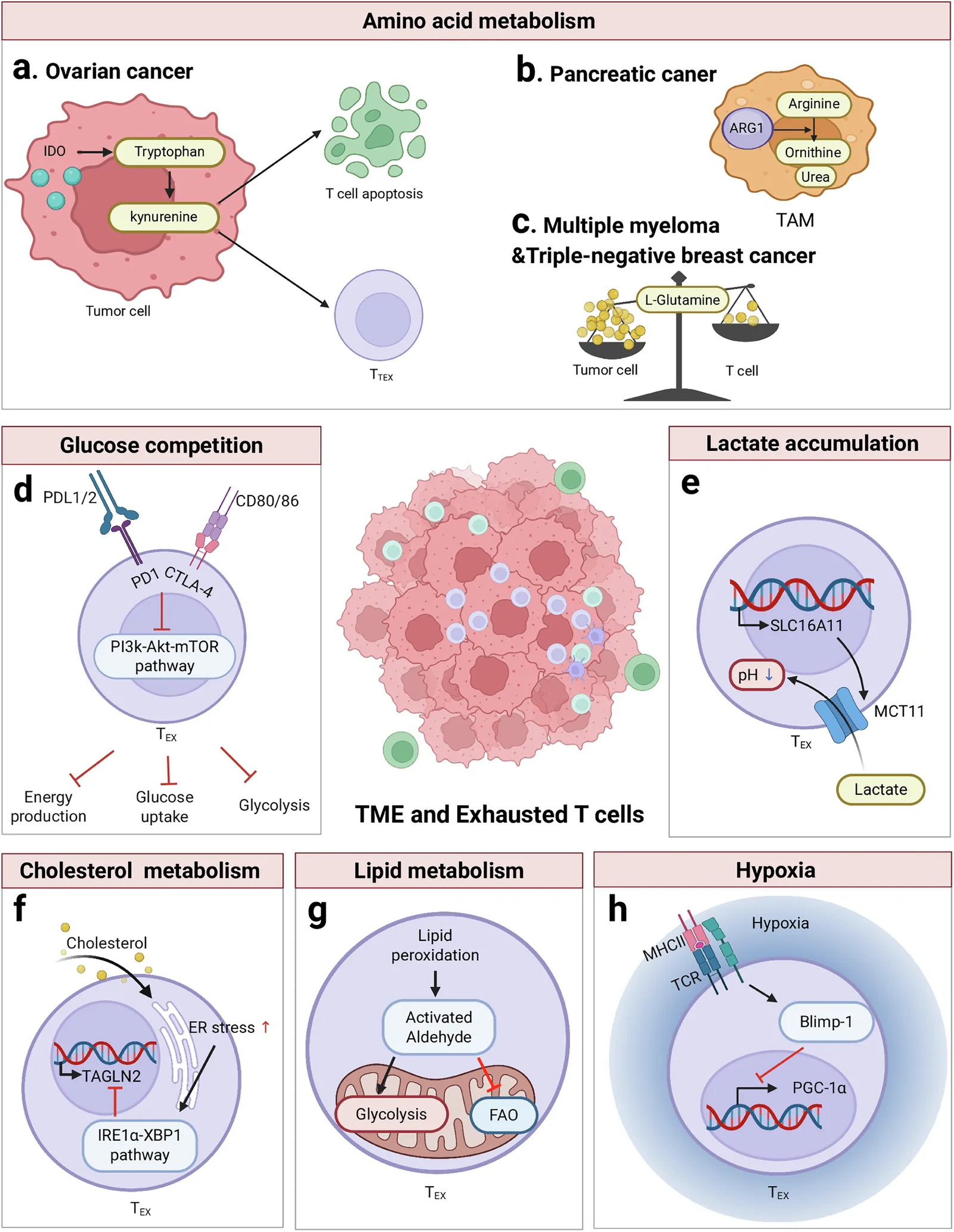
Metabolic constraints on exhausted T cells within the tumour microenvironment. Metabolic competition and toxic metabolite accumulation within the tumour microenvironment (TME) impair the fitness and function of exhausted CD8^+^ T cells. Nutrient availability is reshaped by tumour and stromal cells across different malignancies. **a** In ovarian cancer, tumour cell-expressed IDO depletes tryptophan and generates kynurenine, promoting T cell apoptosis and exhaustion. **b** In pancreatic cancer, tumour-associated macrophages (TAMs) express ARG1 to catabolize arginine, thereby limiting its availability to T cells. **c** In multiple myeloma and triple-negative breast cancer, tumour cells outcompete T cells for L-glutamine, creating a nutrient-restricted state. **d** PD-1 and CTLA-4 signalling suppresses the PI3K–AKT–mTOR pathway, thereby reducing glucose uptake, glycolysis and energy production in exhausted T cells. **e** Elevated extracellular lactate is imported through SLC16A11 (MCT11), further contributing to metabolic dysfunction. **f** Excess cholesterol induces ER stress and activates the IRE1α–XBP1 pathway, promoting exhaustion-associated programs while suppressing TAGLN2. **g** Lipid peroxidation generates reactive aldehydes that inhibit both glycolysis and fatty acid oxidation (FAO). **h** Hypoxia, together with chronic T cell receptor signalling, induces BLIMP1, which represses PGC-1α and impairs mitochondrial fitness, thereby reinforcing T cell exhaustion. The images were created using BioRender (https://biorender.com).

Tumour cells not only compete with T cells from amino acids but also metabolize them into immunosuppressive metabolites that have been shown to reshape T cell metabolic programs and potentially promote exhaustion-associated epigenetic states. Activation of indoleamine 2,3-dioxygenase (IDO) in ovarian tumour cells depletes tryptophan in the TME, suppressing CD8^+^ T cell proliferation. In parallel, kynurenine produced through tryptophan catabolism has been reported to induce T cell apoptosis and may promote exhaustion programs^24^ (Fig. 1a). Conversely, restoring tryptophan availability through IDO inhibition or tryptophan supplementation activates the WARS–TRIP12 pathway, which suppresses PD-1 expression and partially restores T cell function^25^.

Arginine metabolism is increasingly recognized as another critical metabolic checkpoint. In pancreatic cancer models, TAMs secrete arginase 1 (ARG1), which breaks down extracellular arginine and limits T cell response^26^ (Fig. 1b). Pharmacological inhibition of arginase using CB-1158 enhances T cell infiltration and improves the therapeutic efficacy of anti-PD-1 therapy. Similarly, glutamine depletion caused by tumour growth has been shown to impair T cell function in multiple myeloma and triple-negative breast cancer models^27,28^ (Fig. 1c).

#### Glucose competition

Effector T cells, which orchestrate the immune response, rapidly generate energy through aerobic glycolysis, a metabolic program that converts glucose into pyruvate and subsequently lactate, even in the presence of oxygen^29^. However, within the TME, tumour cells consume large amounts of glucose through the Warburg effect, which may contribute to reducing glucose availability for effector T cells. This metabolic competition is further exacerbated by abnormal tumour vasculature, which limits nutrient delivery and potentially places T cells in an energy-depleted state^30,31^. In addition, inhibitory receptors such as PD-1 and CTLA-4 have been shown to suppress T cell metabolic fitness by dampening the PI3K–Akt–mTOR signaling pathway, which regulates glucose uptake and glycolysis^32^ (Fig. 1d). However, direct in vivo measurement of cell-specific glucose uptake within the TME suggested that glucose may not be substantially limiting, and that nutrient uptake is regulated by cell-intrinsic metabolic programs rather than competition between T cells and cancer cells^33^. Furthermore, in vitro mechanistic studies have shown that mitochondrial dysfunction, rather than glucose depletion, may be a primary driver of T cell exhaustion, as chronically stimulated T cells maintain high glycolytic activity yet exhibit progressive oxidative phosphorylation impairment, leading to reactive oxygen species (ROS)-driven activation of exhaustion-associated transcriptional programs^34^.

#### Lactate accumulation

The glycolytic metabolism of tumour cells often results in the accumulation of large quantities of lactate within the TME. Elevated extracellular lactate can disrupt T cell metabolic homeostasis by limiting lactate export through monocarboxylate transporters such as MCT-1, thereby potentially suppressing glycolytic flux and diminishing effector functions^35^. Recent studies further reveal that T_TEX_ cells upregulate SLC16A11, which encodes the monocarboxylate transporter MCT11. Increased MCT11 expression facilitates lactic import into T cells, which may lead to intracellular acidification and metabolic dysfunction^36^ (Fig. 1e).

#### Cholesterol and lipid metabolism

Cholesterol accumulated within the TME excessively enters CD8^+^ T cells, which may induce ER stress, which has been shown to activate the key transcription factor XBP1. Activated XBP1 directly binds to the promoters of *PD-1* and *2B4* genes, upregulating their transcription and thereby contributing to T cell exhaustion^37^. Especially, the IRE1α–XBP1s signalling pathway activated by ER stress suppresses the expression of the cytoskeletal regulator TAGLN2. This impairs FABP5-mediated lipid uptake and mitochondrial fatty acid oxidation (FAO), ultimately compromising T cell function^38^ (Fig. 1f). PD-1, the primary marker of T cell exhaustion, enhances CPT1A expression to promote FAO, whereas the lipase ATGL induces lipolysis to secrete fatty acids. It has been widely reported that T cells shifting their energy production from glycolysis to FAO exhibit diminished effector functions but enhanced longevity within the TME, thereby facilitating the anti-tumour efficacy of ICB^39^. However, recent studies have revealed that active aldehydes generated by lipid peroxidation and accumulated in CD8^+^ T cells form a metabolic toxicity loop. This loop has been suggested to impair FAO by damaging mitochondrial structure, thereby potentially trapping T cells in an inefficient glycolysis-dependent state^40^ (Fig. 1g).

#### Hypoxia

Hypoxia is a hallmark of most solid tumours and arises from rapid tumour growth combined with disorganized vascular architecture that limits oxygen delivery to the TME^41^. In addition, high metabolic demands of proliferating tumour cells further exacerbate oxygen depletion. Recent studies indicate that hypoxia alone is not sufficient to induce T cell exhaustion^42^. Rather, exhaustion appears to be exacerbated when chronic T cell receptor (TCR) stimulation occurs together with hypoxic stress. Under hypoxic conditions, mitochondrial adaptation is normally supported by the transcriptional coactivator PGC-1α. However, persistent TCR signalling induces the transcription factor Blimp-1, which suppresses PGC-1α expression^42^ (Fig. 1h). This disruption can lead to mitochondrial dysfunction, elevated ROS production and activation of NFAT signalling pathways that may promote exhaustion programs^42^. Conversely, mitochondrial dysfunction itself can drive exhaustion independently of hypoxia^43^. Deletion of the mitochondrial phosphate carrier SLC25A3 induces mitochondrial stress and glycolytic reprogramming even under normoxic conditions, promoting differentiation of T_PEX_ into T_TEX_. These findings suggest that metabolic and mitochondrial stress, rather than hypoxia alone, may represent key drivers of exhaustion differentiation.

## Defining and measuring T cell exhaustion

### Hallmarks of T cell exhaustion

Phenotypically, exhausted T cells are characterized by sustained expression of inhibitory immune-checkpoint receptors, progressive loss of effector cytokine production and cytotoxic activity, metabolic rewiring, and a distinct transcriptional and epigenetic landscape^44–48^. This state is driven by persistent antigen-dependent signalling initiated during T cell priming and reinforced by repeated antigen encounter. Inhibitory receptors including PD-1, CTLA-4, TIM-3, LAG-3 and TIGIT are progressively upregulated, whereas effector function declines in a hierarchical loss of IL-2 production followed by TNF and IFNγ^44,49,50^. Metabolically, exhausted T cells exhibit mitochondrial dysfunction, reduced glycolytic capacity and impaired mTOR signalling, reflecting a state of cellular energy insufficiency^42,46,51^.

**Fig. 2:**
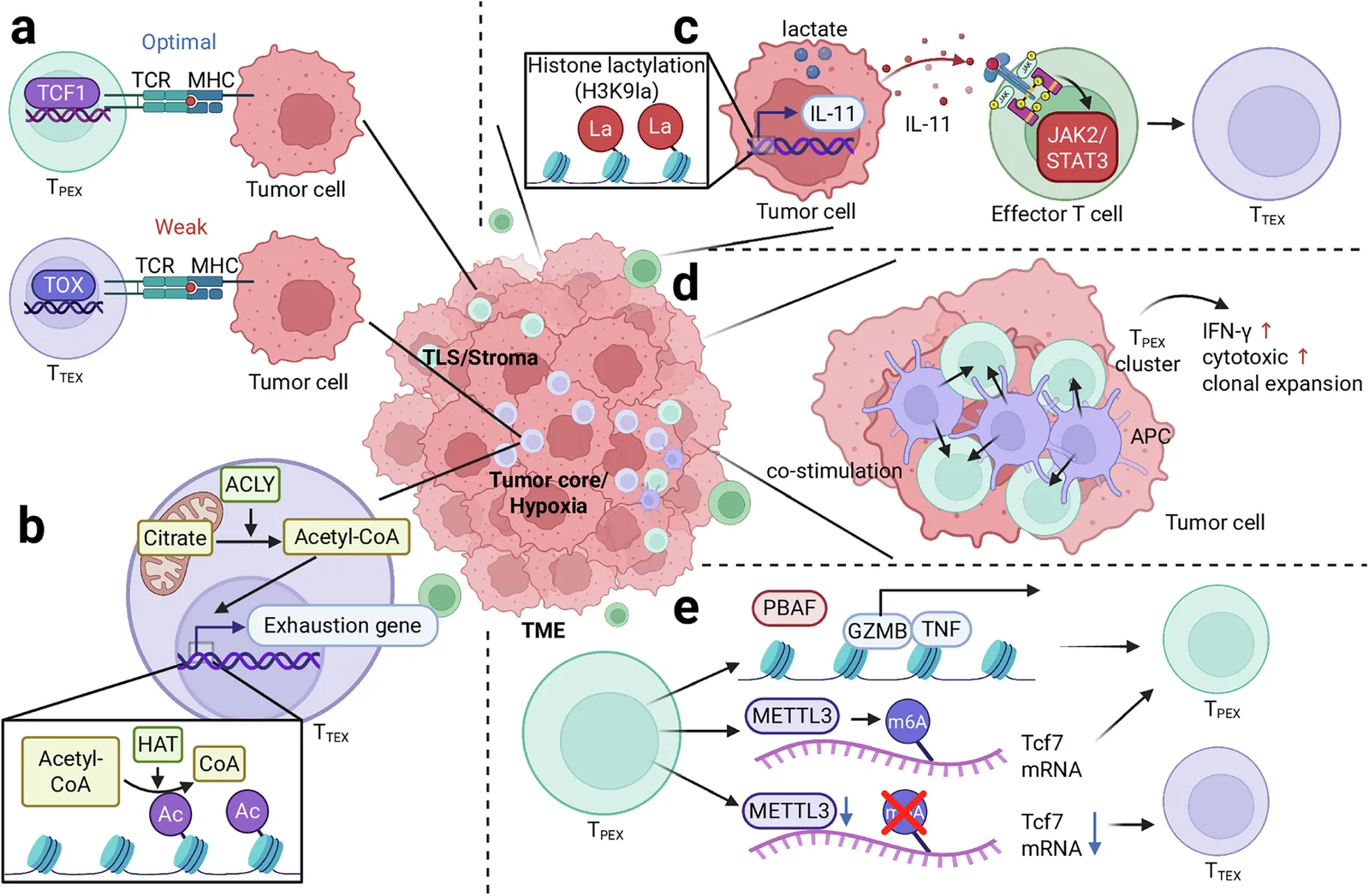
Integrated regulation of progenitor exhausted CD8^+^ T cells in the tumour microenvironment. **a** T cell receptor (TCR) signal strength influences CD8^+^ T cell fate in tumours. Optimal TCR–MHC interactions support maintenance of TCF1^+^ progenitor exhausted T cells (T_PEX_), whereas weak TCR signalling promotes induction of TOX and differentiation towards terminally exhausted T cells (T_TEX_). **b** In the metabolically stressed tumour microenvironment (TME), citrate-derived acetyl-CoA production through ACLY supports histone acetylation via histone acetyltransferases (HATs), thereby promoting transcriptional programs associated with exhaustion-related gene expression. **c** Tumour-derived lactate induces histone lactylation (for example, H3K9la) and enhances IL-11 expression, which activates JAK2–STAT3 signalling in effector T cells and contributes to their dysfunction and progression towards exhaustion. **d** Spatially organized T_PEX_ clusters in association with antigen-presenting cells (APCs) provide local co-stimulatory signals that promote IFNγ production, cytotoxic function and clonal expansion, thereby supporting anti-tumour CD8^+^ T cell responses. **e** Epigenetic and post-transcriptional regulation further controls T_PEX_ maintenance. PBAF restrains chromatin accessibility at effector loci such as GZMB and TNF, whereas METTL3-dependent m6A modification stabilizes Tcf7 mRNA to preserve the T_PEX_ state. By contrast, loss of METTL3 reduces Tcf7 mRNA stability and favours differentiation into T_TEX_. The images were created using BioRender (https://biorender.com).

Within the exhausted CD8^+^ T cell compartment, two principal subsets have been defined. Differences in TCR signalling quality contribute to exhaustion-state heterogeneity, with optimal TCR signalling favouring maintenance of TCF1^+^ T_PEX_ and weaker or dysregulated signalling promoting differentiation towards terminally exhausted cells^52^ (Fig. 2a). These include T_PEX_ and T_TEX_. T_PEX_ cells are characterized by TCF-1^+^PD-1^+^CXCR5^+^ phenotypes and retain self-renewal capacity while continuously generating downstream exhausted populations, although they display limited immediate cytotoxicity^47,53^. By contrast, TCF-1^−^ T_TEX_ cells exhibit higher TOX expression and severely restricted proliferative capacity, and represent a near-terminal differentiated state^47,53,54^. However, exhausted CD8^+^ T cell differentiation is not restricted to a simple T_PEX_–T_TEX_ dichotomy. A four-stage developmental hierarchy has been described in which TCF1^+^ progenitor exhausted subsets progress through an intermediate exhausted state, termed Tex^int^, before terminal exhaustion. Tex^int^ cells are TCF1^−^ and T-bet-high, re-engage effector-like and cytotoxic programs, and exhibit relatively lower TOX expression than progenitor and terminal exhausted subsets. During the transition from Tex^int^ towards terminal exhaustion, TOX expression increases again, indicating that TOX expression is dynamically regulated across exhausted T cell subsets rather than following a simple low-to-high linear pattern^55^. In addition, recent single-cell multiomics analyses have shown that Tex^int^ can act as a bifurcation point toward either T_TEX_ cells or effector-like Tex^KLR^ cells. Tex^KLR^ cells are characterized by KLR/NK receptor-associated and cytotoxic programs, whereas T_TEX_ cells display stronger terminal exhaustion signatures^56^. Importantly, these subsets occupy distinct spatial niches within tumours. T_PEX_ cells are enriched in tumour-draining lymph nodes and preferentially localize to tertiary lymphoid structures rather than the tumour parenchyma^4,44,57^. By contrast, T_TEX_ cells accumulate in tumour nests or hypoxic stromal regions enriched in dysfunctional antigen-presenting cells and immunosuppressive signals derived from cancer-associated fibroblasts^58^. Hypoxia-driven metabolic rewiring, marked by increased ACLY and decreased ACSS2 expression, enhances citrate-derived acetyl-CoA production and KAT2A-mediated histone acetylation, thereby promoting exhaustion-related gene expression and terminal T cell exhaustion^59^ (Fig. 2b) In addition, in head and neck squamous cell carcinoma, lactate-driven histone lactylation, particularly H3K9la, induces IL-11 expression, which activates JAK2/STAT3 signalling and promotes immune-checkpoint gene transcription in CD8^+^ T cells, thereby driving functional suppression and terminal exhaustion^60^ (Fig. 2c) High-resolution imaging analyses have revealed that tumour-reactive CD8^+^ T cells form heterotypic clusters with tumour cells or antigen-presenting cells, and these clustered T cells exhibit enhanced exhaustion-associated features and signatures of metabolic stress^61^ (Fig. 2d). These observations underscore the critical role of stromal and metabolic constraints in maintaining and reinforcing exhaustion states^58^.

At the transcriptional and epigenetic levels, chronic antigen stimulation engages regulatory circuits centred on the NFAT–TOX–NR4A axis^62^. These programs are reinforced by epigenetic remodelling, including DNMT3A-mediated DNA methylation and histone modifications, which restrict accessibility of effector gene loci while maintaining accessibility at exhaustion-associated regulatory regions^63^. Epigenetic and post-transcriptional regulation further contribute to exhaustion-state heterogeneity, as PBAF restricts chromatin accessibility at effector-associated loci, whereas METTL3-mediated m6A modification stabilizes Tcf7 mRNA and supports maintenance of T_PEX_ cells; by contrast, loss of METTL3 reduces Tcf7 expression and facilitates progression towards T_TEX_^64^ (Fig. 2e). As a result, terminal exhaustion becomes an epigenetically encoded state with limited reversibility that can be stably propagated through cell division^1,2,65^. Upon TCR-mediated activation, CD8^+^ T cells proliferate and diverge into two principal lineages. These include KLRG1^low^ memory precursor effector cells (MPECs) and KLRG1^high^ short-lived effector cells^66^. MPECs possess long-term survival capacity and retain the potential to differentiate into memory T cells whereas short-lived effector cells exhibit potent effector function but have a short life and decline during the contraction phase^66^. In acute or resolved immune responses, these precursors are represented by memory precursor T cells, often referred to as MPECs, which preferentially give rise to memory T cells^67^. By contrast, during chronic antigen stimulation, T_PEX_ cells represent antigen-experienced TCF1^+^ precursor populations that express exhaustion-associated markers and continuously replenish downstream exhausted T cells. Although T_PEX_ cells share stemness-associated properties with memory-associated populations, including MPECs and T stem cell memory (T_SCM_) cells, such as self-renewal, proliferative capacity and TCF1-associated transcriptional programs, these populations are not equivalent^67,68^. MPECs and T_SCM_ cells belong primarily to memory-associated lineages, whereas T_PEX_ cells are chronically stimulated, transcriptionally and epigenetically distinct, and committed to the exhausted lineage^68^. Therefore, when describing progenitor-like CD8^+^ T cell states, T_PEX_ cells should be distinguished from memory-derived populations such as MPECs and T_SCM_ cells, despite their overlapping stemness-associated features.

### Confounding states in interpreting T cell exhaustion

Accurate interpretation of T cell exhaustion in tumours is frequently complicated by biological states that share overlapping phenotypic or transcriptional features with exhausted T cells. Several distinct contexts can therefore confound the identification and functional interpretation of exhausted T cell populations.

A first source of confusion arises from the overlap between T cell activation and early exhaustion programs. Historically, effector and exhausted T cells were distinguished largely on the basis of PD-1 expression levels^69^. However, exhaustion is now understood as a differentiation trajectory that progresses from T_PEX_ to T_TEX_ cells. Although T_TEX_ cells are readily identifiable owing to their limited proliferative capacity and impaired cytokine production, T_PEX_ cells retain partial effector function and active proliferative potential, making them difficult to distinguish from conventional effector T cells based solely on inhibitory receptor expression. Importantly, despite comparable PD-1 expression levels, only T_PEX_ cells are capable of responding to ICB^44^, highlighting the need to distinguish these populations accurately. Emerging evidence further indicates that T_PEX_ cells arise early during T cell activation rather than being generated through progressive differentiation from effector T cells under chronic antigen exposure^52,70^. Consequently, effector and T_PEX_ cells may share early transcriptional and phenotypic factures, including PD-1 expression, complicating their discrimination using single-marker approaches^71^. For example, integration of TCF1, PD-1 and CD8 expression has been used to define colorectal cancer. Notably, increased frequencies of T_PEX_ cells correlate with improved colorectal cancer patient survival and enhanced responsiveness to immunotherapy^72^.

A second factor derives from the coexistence of bystander tumour-infiltrating lymphocytes (TILs) and tumour antigen-specific T cells. Tumour tissues contain heterogenous TIL populations, including T cells that have never encountered tumour antigens, as well as tumour-reactive clones undergoing chronic antigen stimulation^73^. Bystander T cells can become activated through inflammatory cytokines present in the TME, including type I interferons, IL-12, IL-15 and IL-18, even in the absence of TCR signalling^74,75^. As a result, these cells can acquire activation phenotypes resembling tumour-specific T cells while remaining mechanistically distinct from exhausted populations targeted by ICB therapy. Although CD39 expression has been proposed as a surrogate marker of chronic antigen exposure, substantial inter-patient variability limits its utility as a single discriminating marker^76^. Integrative approaches that combine CD39 with transcriptional markers, such as CXCL13 and TOX, together with single-cell transcriptomic analysis, therefore provide a more reliable framework for distinguishing tumour-specific exhausted T cells from cytokine-activated bystander populations.

A third challenge arises from the phenotypic overlap between tissue-resident memory T (T_RM_) cells and exhausted T cells. T_RM_ cells represent non-circulating memory T cells that reside in peripheral tissues and typically express surface markers including CD69 and CD103 (ref.^77^). However, recent studies have demonstrated that exhausted T cells can adopt components of the tissue residency program to persist within tumour tissues^78,79^. Both populations express overlapping transcriptional signatures, including ZNF683 and CXCR6, and downregulate migration-associated genes such as KLF2, S1PR1 and S1PR5 (ref.^78,79^). Consequently, classification strategies relying solely on canonical T_RM_ markers may misidentify exhausted T cells as resident memory populations. Functional context therefore becomes essential for accurate interpretation, as T_RM_ cells generally represent bystander populations activated independently of chronic antigen stimulation, whereas exhausted T cells arise in response to persistent antigen exposure^76^. This distinction has important clinical implications because T_RM_ cells are often associated with a favourable prognosis, whereas ICB responsiveness primarily depends on the presence of tumour-specific exhausted T cell populations.

Finally, spatial context within the TME represents an additional source of analytical bias. High-dimensional imaging approaches such as CODEX have revealed that tumours are organized into discrete cellular neighbourhoods composed of distinct immune and stromal networks^80^. As a result, T cells with similar phenotypic signature can exhibit markedly different functional states depending on the microenvironmental niche in which they reside^80^. For example, tumour cores often characterized by chronic antigen exposure, hypoxia and metabolite accumulation frequently harbour T_PEX_, whereas tumour margins may contain effector-like T cells that have only recently encountered antigens. These findings highlight the importance of integrating spatial information when interpreting exhaustion states within tumours.

### Experimental framework for defining T cell exhaustion

T cell exhaustion cannot be defined by a single marker. As discussed above, expression of inhibitory receptors such as PD-1, TIM-3 and LAG-3 can also occur during transient T cell activation, and effector T cells may exhibit temporary functional impairment under certain conditions. In addition, bystander T cells and tissue-resident T cells within the TME can share overlapping phenotypic features with exhausted T cells. Consequently, surface marker-based definitions alone are insufficient to accurately delineate the exhaustion lineage.

Analysis of TCR clonality provides an important complementary dimension. Clonal expansion reflects differentiation histories shaped by repeated antigen exposure and supports the concept that exhaustion represents an antigen-experienced and lineage-stabilized state rather than a transient hypofunctional condition^81^. Recent advances in single-cell technologies, including scRNA-seq, CyTOF, ATAC-seq, multiome profiling and TCR-seq, together with spatial approaches such as spatial transcriptomics and imaging mass cytometry, now enable exhaustion to be examined through a multilayered framework integrating phenotype, function and regulatory state^82–84^. For example, droplet-based scRNA-seq has identified distinct intratumoural exhausted subsets defined by exhaustion-associated transcriptional programs, whereas imaging mass cytometry has spatially validated these immune ecosystems within tumour tissues^84^. Complementary analyses of TLS further indicate that exhausted T cells can be embedded within organized immune niches shaped by stromal and myeloid networks, which may influence responsiveness to immunotherapy^84^.

Conceptual frameworks have therefore been proposed to define exhaustion using multidimensional criteria. One such framework, termed Metabolic, Epigenetic, Transcriptional, Activation-based phenotypic markers (M.E.T.A.)^85^, emphasizes that inhibitory receptor expression or functional impairment alone is insufficient to define exhaustion. Instead, exhaustion should be identified through integrated signatures centred on TOX-associated transcriptional programs and supported by metabolic, epigenetic and activation states. Building on these principles, minimal criteria for defining exhaustion should incorporate sustained antigen exposure, persistent combinatorial inhibitory receptor expression, transcriptional regulatory networks, functional hierarchy, metabolic adaptation and epigenetic stabilization, together with TCR context where antigen specificity can be inferred^81,85^.

At the transcriptional level, exhausted T cells are characterized not only by differential transcription factor expression but also by the distinct regulatory architecture. Dominance of NFAT-driven transcriptional programs, activation of TOX–NR4A circuits and reduced T-bet activity characterize exhausted states^62^. In particular, the balance between NFAT and AP-1 signalling appears to represent a critical checkpoint distinguishing effector differentiation from exhaustion^86^. Metabolically, naïve T cells primarily rely on oxidative phosphorylation, whereas effector T cells utilize aerobic glycolysis^87^. In the TME, metabolic competition can restrict glucose availability for tumour-infiltrating T cells, reducing mTOR activity, glycolysis and IFNγ production^88^. However, metabolic adaptations vary across exhausted T cell subsets and states, precluding a uniform reliance on FAO or the suppression of glycolysis. Persistent antigen stimulation can impair mitochondrial oxidative phosphorylation and substrate oxidation, thereby limiting ATP production, proliferation and self-renewal^34^. These metabolic features further distinguish exhausted populations from effector and memory T cells and should therefore be incorporated into defining criteria. Persistent antigenic stimulation represents another essential determinant of exhaustion. Experimental evidence shows that PD-1 blockade induces only transient functional reinvigoration in antigen-rich environments, after which T cells revert to an exhausted state^89^. Exhaustion signatures are therefore typically associated with sustained co-expression of multiple inhibitory receptors, including PDCD1 (PD-1), ENTPD1 (CD39), LAG3, HAVCR2 (TIM-3), ITGAE (CD103), CXCL13 and TNFRSF9 (4-1BB), highlighting the limitations of relying on PD-1 expression alone^5^. Finally, exhausted T cells display a distinct epigenetic architecture. Chromatin accessibility remains persistently open at loci encoding inhibitory receptors and exhaustion-associated transcription factors, whereas loci associated with effector function and metabolic plasticity become relatively inaccessible. Stabilized enhancer networks together with DNA methylation reinforce this transcriptional configuration, establishing an epigenetically fixed exhaustion program^65^.

Taken together, accurate assignment of exhaustion states in tumours requires an integrative framework rather than reliance on any single metric. Phenotypic markers, functional capacity, transcriptional programs, metabolic state, epigenetic configuration, TCR clonality and spatial context each provide complementary information. Integration of these dimensions enables tumour-infiltrating T cells to be positioned along defined exhaustion trajectories and distinguished from transient activation, bystander responses or tissue residency.

## Immunotherapy and T cell exhaustion

### Reinvigoration of exhausted T cells by immune-checkpoint blockade

Tumour-specific CD8^+^ T cells progressively acquire exhaustion as a consequence of chronic antigen stimulation and the immunosuppressive TME. This process is characterized by sustained expression of inhibitory checkpoint receptors, including PD-1, CTLA-4, VISTA, TIGIT, TIM-3 and LAG-3. Immune-checkpoint inhibitors (ICIs) block these inhibitory pathways and thereby restore anti-tumour immunity by partially reinvigorating exhausted T cells^90,91^.

Despite durable clinical responses in subsets of patients, the majority of individuals with advanced cancers fail to achieve sustained benefit from ICIs. Clinically, resistance to checkpoint blockade can manifest as primary resistance (lack of initial response), adaptive resistance (loss of response after initial benefit), or acquired resistance following tumour relapse. Even when responses occur, reinvigoration of tumour-specific CD8^+^ T cells after PD-1 blockade is frequently transient, and many cells subsequently progress towards terminal exhaustion^89,92,93^. Increasing evidence indicates that resistance to ICIs reflects a complex network of mechanisms involving exhausted T cell differentiation, defective costimulatory signalling, impaired antigen presentation, disrupted interferon signalling, non-inflamed TMEs and metabolic constraints^94^.

Importantly, ICB does not directly restore the function of terminally exhausted CD8^+^ T cells. Instead, anti-PD-1 therapy preferentially expands TCF1^+^ T_PEX_ cells and promotes their differentiation into new effector-like exhausted progeny, thereby mediating transient anti-tumour responses^44^. This mechanism explains why clinical responses to PD-1 blockade often depend on the presence of a progenitor exhausted T cell pool.

### Multi-checkpoint targeting along the exhaustion differentiation trajectory

Persistent resistance following PD-1/PD-L1 or CTLA-4 blockade is frequently associated with co-expression of additional inhibitory receptors such as LAG-3, TIM-3 and TIGIT. Consequently, combinatorial checkpoint blockade strategies have emerged as an approach to enhance T cell reinvigoration and overcome resistance mechanisms^95^. For example, relatlimab directly targets the ligand-binding interface of LAG-3 and blocks its interaction with both MHC class II and FGL1, thereby preventing sustained inhibitory signalling within the TME. When combined with PD-1 blockade, two distinct regulatory axes are simultaneously relieved: TCR signalling inhibition mediated by PD-1 and ligand-dependent suppression mediated by LAG-3. This dual inhibition restores IL-2 production and enhances T cell activation in LAG-3^+^ T cells^96^. Single-cell transcriptomic analyses further demonstrate that PD-1–LAG-3 dual blockade does not erase exhaustion-associated epigenetic programs but instead expands effector-like CD8^+^ T cells that retain exhaustion features while expressing cytotoxic gene modules such as IFNG, PRF1 and GZMB. These findings indicate that checkpoint blockade primarily promotes the generation and expansion of functional progeny derived from exhausted T cell pools rather than complete reversal of terminal exhaustion^97^. Similarly, TIM-3 can function as a residual inhibitory pathway following PD-1 blockade. Combined TIM-3–PD-1 inhibition further augments T cell activation and anti-tumour immunity. In patients with non-small cell lung cancer (NSCLC) who progressed on prior PD-(L)1 therapy, combination treatment with the anti-TIM-3 antibody cobolimab and the anti-PD-1 antibody dostarlimab induced objective responses and disease control with manageable toxicity, particularly in tumours exhibiting high baseline TIM-3 expression^98^.

Complementary effects are also observed with PD-1 and CTLA-4 blockade. Single-cell transcriptomic and TCR tracking studies in melanoma reveal that CTLA-4 blockade preferentially expands progenitor exhausted CD8^+^ T cells expressing TCF7, CCR7 and SELL, whereas PD-1 blockade promotes differentiation of these progenitors into terminally differentiated exhausted T cells expressing TOX, PDCD1 and EOMES. Combination therapy simultaneously expands the progenitor pool and drives effector differentiation, resulting in greater magnitude and durability of anti-tumour CD8^+^ T cell responses^99^.

### Reprogramming the tumour microenvironment and terminally exhausted T cells to enhance checkpoint therapy

**Fig. 3:**
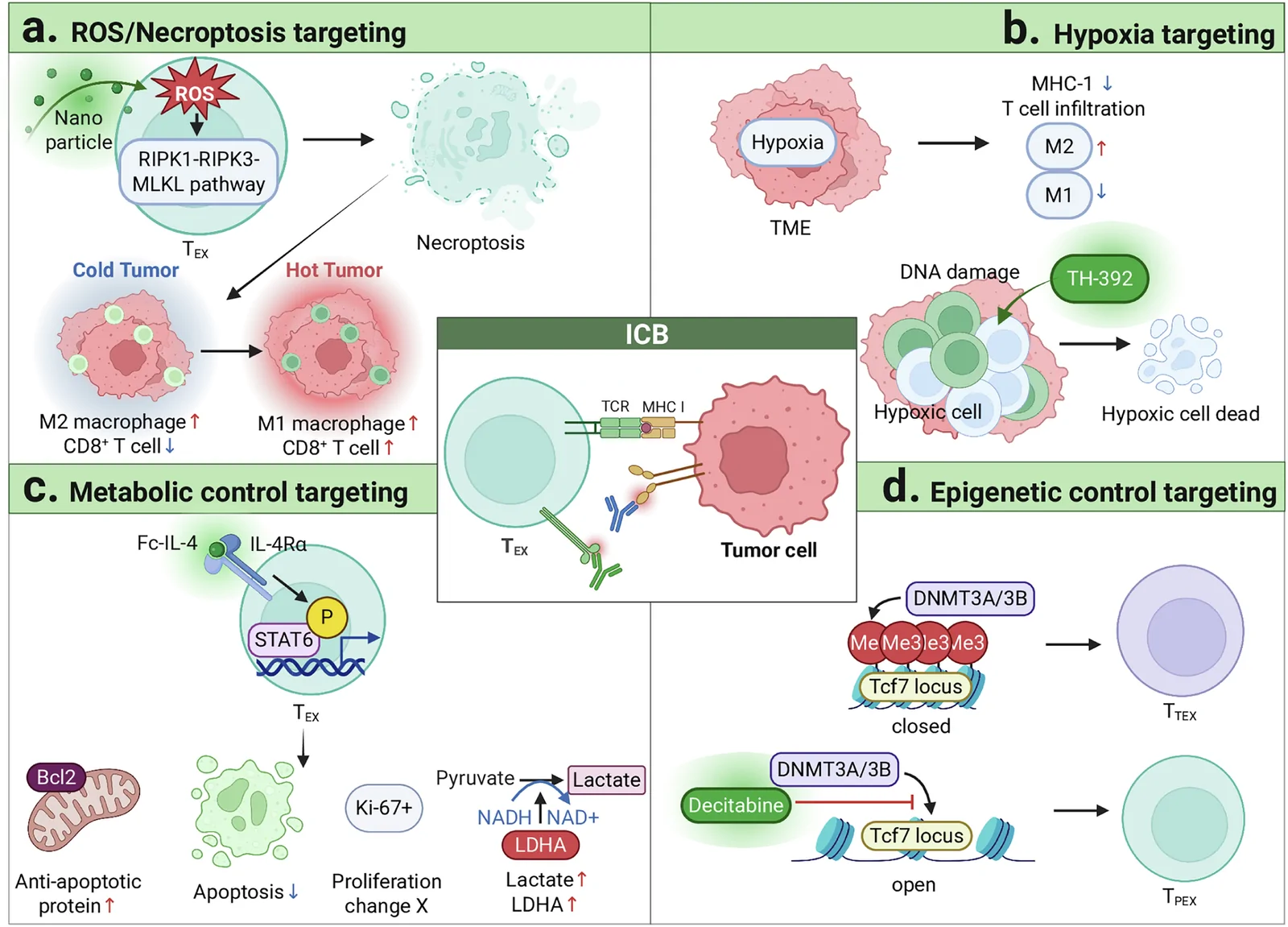
Therapeutic strategies for overcoming T cell exhaustion and improving responsiveness to immune-checkpoint blockade. **a** Nanoparticle-induced reactive oxygen species (ROS) activate the RIPK1–RIPK3–MLKL pathway, promoting necroptosis and inflammatory remodelling of the tumour microenvironment (TME). This process contributes to conversion of cold tumours into hot tumours, accompanied by decreased M2-like macrophages and increased M1-like macrophages and CD8^+^ T cells. **b** Hypoxia in the TME is associated with reduced MHC class I expression, impaired T cell infiltration, increased M2-like macrophages and reduced M1-like macrophages. The hypoxia-targeting agent TH-392 induces DNA damage and selective killing of hypoxic tumour cells, thereby alleviating hypoxia-driven immune suppression. **c** Fc–IL-4 engages IL-4Rα and activates STAT6 signalling in exhausted T cells, promoting survival-associated programs, increasing anti-apoptotic proteins, reducing apoptosis and altering proliferative responses. This pathway is also associated with enhanced LDHA expression and lactate production, indicating metabolic rewiring in T_EX_ cells. **d** Epigenetic regulation of the Tcf7 locus influences exhausted T cell fate. DNMT3A/3B-mediated repression closes the Tcf7 locus and promotes differentiation towards terminally exhausted T cells (T_TEX_), whereas decitabine counteracts this repression, maintains an open chromatin state and supports the progenitor exhausted T cell (T_PEX_) state. The images were created using BioRender (https://biorender.com).

Beyond multi-checkpoint blockade, increasing attention has focused on therapeutic strategies that reshape the immunosuppressive TME. Metabolic and hypoxic constraints represent major barriers to effective T cell reinvigoration and therefore constitute important targets for combination therapy. For example, inhibition of glutamine metabolism using GLS1-targeted nanoplatforms reduces immunosuppressive macrophage populations while promoting inflammatory macrophage polarization. When combined with anti-PD-L1 therapy, this metabolic intervention enhances immunogenic cell death, increases CD8^+^ T cell infiltration and significantly suppresses tumour growth^100^ (Fig. 3a). Hypoxia also contributes to resistance to checkpoint blockade. ICI-resistant tumours frequently exhibit increased hypoxia, reduced infiltration of CD8^+^ and CD4^+^ T cells and diminished interferon signalling. Hypoxic conditions suppress tumour-intrinsic MHC class II expression, thereby impairing anti-tumour immunity. Pharmacological alleviation of tumour hypoxia using hypoxia-targeted agents restores antigen presentation, increases T cell infiltration and improves responsiveness to the checkpoint blockade^101^ (Fig. 3b).

Emerging evidence further suggests that terminally exhausted CD8^+^ T cells are not irreversibly fixed end states. Type 2 cytokine signalling can directly act on T_PEX_ through IL-4Rα–STAT6 pathways to enhance survival and metabolic fitness. These signals activate PI3K–AKT–mTOR pathways, increase glycolysis and NAD^+^ regeneration, suppress apoptosis and restore cytotoxic molecule production, including granzyme B and IFNγ. As a result, functionally impaired T_PEX_ regain anti-tumour activity, and, in combination with adoptive T cell therapy, ICIs or CAR-T approaches, promotes tumour clearance and durable immune memory^102^ (Fig. 3c).

In addition, epigenetic priming with low-dose decitabine can further enhance checkpoint efficacy by promoting the expansion and functional reinvigoration of progenitor exhausted CD8^+^ T cells. In tumour models, decitabine combined with anti-PD-1 therapy increased the abundance and clonal expansion of PD-1^+^TCF1^+^T_PEX_, reprogrammed their transcriptional and epigenetic landscape, sustained JunD/AP-1 activity and limited terminal differentiation, thereby improving anti-tumour responses^93^ (Fig. 3d).

Collectively, these findings indicate that maximizing the efficacy of checkpoint blockade requires moving beyond single-receptor inhibition towards integrated therapeutic strategies that simultaneously target multiple stages of the exhaustion differentiation trajectory and the suppressive features of the TME. Multi-checkpoint blockade, metabolic reprogramming, hypoxia-targeted therapies and cytokine-mediated reinforcement of T cell survival operate in complementary ways to sustain anti-tumour immunity. This integrated perspective reframes exhausted T cells not simply as dysfunctional cells that must be rescued from inhibition but as dynamic populations that can be selectively expanded, reprogrammed and redirected towards effective anti-tumour responses. Understanding how therapeutic interventions shape exhaustion trajectories therefore represents a critical frontier for the development of next-generation cancer immunotherapies.

## Chimeric antigen receptor T cell exhaustion in solid tumours

Growing insight into the mechanisms of T cell exhaustion has prompted efforts to apply these findings to adoptive cell therapy. Because T cells for adoptive cell therapy are generated in vitro, it is possible to confer resistance to exhaustion at this stage. In particular, for chimeric antigen receptor (CAR) T cell therapy, various in vitro reprogramming strategies targeting the transcriptional, epigenetic and metabolic factors of exhaustion are being developed. Although CAR T cell therapy has achieved remarkable clinical success in haematological malignancies, its efficacy in solid tumours remains limited^103^. To date, no CAR T therapies have received FDA approval for solid tumours. Major barriers include the physical and metabolic constraints imposed by the TME, persistent antigen stimulation and progressive T cell exhaustion. Consequently, current engineering strategies increasingly focus on enhancing the functional persistence, metabolic fitness and exhaustion resistance of CAR T cells.

T cell exhaustion is associated with the dominance of inhibitory AP-1 and IRF transcriptional complexes that remodel chromatin and suppress IL-2 production. One strategy for counteracting this program involves engineering CAR T cells to overexpress the transcription factor c-Jun. c-Jun overexpression displaces inhibitory AP-1/IRF complexes from chromatin binding sites and restores transcriptional programs associated with T cell activation^104^ (Table 1). This reprogramming enhances IL-2 production and improves anti-tumour activity in solid tumour models such as HER2^+^ osteosarcoma^104^. Importantly, c-Jun-engineered CAR T cells maintain a stem cell memory phenotype and exhibit reduced expression of inhibitory receptors such as PD-1 and CD39, thereby preserving long-term functional persistence even under chronic tonic signalling^104^. The differentiation state of the starting T cell population also critically influences CAR T cell performance^105^. Memory-derived CAR T cells often exhibit strong cytotoxicity but limited proliferative capacity and increased susceptibility to exhaustion compared with naïve-derived cells^105^. Transcriptomic and chromatin accessibility analyses have identified RUNX2 as a key transcription factor driving effector-like differentiation programs^105^. Overexpression of RUNX2 in naïve-derived CAR T cells enhances anti-tumour potency and resistance to exhaustion while preserving self-renewal capacity^105^. Another recent study reveals that the LXRβ–RelB axis is a transformative mechanism for reprogramming CAR T cells to resist exhaustion within the TME^106^. LXRβ functions as a metabolic master regulator that promotes self-replication and metabolic fitness required for long-term survival, whereas RelB was discovered to be a key driver of terminal exhaustion signalling^106^. Engineering CAR T cells to overexpress LXRβ while deleting RelB produced a strong synergistic effect. This dual reprogramming strategy enhances CAR T cell persistence and anti-tumour potency in solid tumours by simultaneously improving metabolic fitness and limiting T cell exhaustion.

**Table 1:** Strategies to overcome chimeric antigen receptor T cell exhaustion.

Category	Target	Modulation	Mechanism	Refs.
Transcriptional regulation	c-Jun	OE	Induction of exhaustion resistance via displacement of immunoregulatory AP-1 complexes (JunB/BATF) from DNA binding sites and restoration of IL-2 production	^104^
	RUNX2	OE	Enhancing effector function and suppressing exhaustion differentiation through chromatin accessibility regulation	^105^
	LXRβ and RelB	LXRβ OE and RelB KO	Synergistic effects of self-replication via LXRβ and blocking exhaustion signalling via RelB deficiency	^106^
Epigenetic remodelling	DNMT3A	KO	Prevention of de novo DNA methylation to maintain the epigenetic accessibility of stemness-associated genes (TCF7, LEF1, CD28) and induce exhaustion resistance	^107^
	TET2	Partial suppression	Promotion of central memory T cell differentiation and long-term survival through epigenetic reprogramming	^108^
	SUV39H1	KO	Prevention of H3K9 methylation to sustain the chromatin accessibility of memory-associated genes and enhance long-term functional persistence	^109^
Metabolic engineering	PRODH2	OE	Enhances metabolic fitness and anti-tumour potency by converting 4Hyp to PHC in the proline metabolic pathway	^110^
	IL-4 signalling (LDHA)	Fc-IL-4 treatment	Reactivates exhausted T cells in an LDHA-dependent manner by normalizing NAD^+^ and glycolytic metabolism through the STAT6–mTOR pathway	^102^
	PGC-1α	Met + Rap pretreatment	Enhances mitochondrial spare respiratory capacity by activating PGC-1α, sustaining anti-tumour activity under hypoxia	^112^
	TAGLN2–FABP5	OE	Preservation of the cytoskeletal factor TAGLN2 to maintain the movement of FABP5 to the cell surface, bypassing tumour-induced ER stress	^111^
	Glut3	OE	Enhances glucose uptake and glycolysis in low-glucose environments to prevent CAR T cell exhaustion	^32^
Signalling and structural adaption	IL-4/IL-7 receptor	Inverting	Enhanced efficacy through IL-4-induced IL-7 signalling and breast cancer-associated MUC1 antigen recognition	^113^
	IL-4	Addition	Enhancing fitness at both transcriptomic and epigenomic levels	^114^

Preventing epigenetic fixation of exhaustion has emerged as another key strategy for sustaining CAR T cell activity. A recent study found that the DNA methyltransferase DNMT3A promotes exhaustion-associated chromatin remodelling in CAR T cells during chronic antigen exposure^107^. Deletion of DNMT3A preserves accessibility of stemness-associated genes as TCF7, LEF1 and CD28, thereby preventing terminal exhaustion and maintaining long-term functional potential. Similarly, modulation of the epigenetic regulator TET2 can influence CAR T cell differentiation. Partial suppression of TET2 promotes development of CAR T cells with central memory-like phenotypes characterized by enhanced self-renewal capacity and long-term persistence^108^. Histone modifiers also contribute to exhaustion stability. For example, SUV39H1-mediated H3K9 methylation represses memory-associated genes and locks CAR T cells into terminal differentiation states^109^. By knocking out SUV39H1, researchers maintained the chromatin accessibility of these memory-associated genes, allowing CAR T cells to resist exhaustion. This modification ensures that the cells remain functional and continue to expand even after repeated challenges within the TME, providing more durable anti-tumour efficacy.

Metabolic reprogramming represents a promising approach to sustaining CAR T cell activity in metabolically hostile TMEs. Screening approaches have identified PRODH2, an enzyme involved in proline metabolism, as a metabolic regulator that enhances CAR T cell persistence and anti-tumour activity^110^. Overexpression of PRODH2 improves CAR T cell function in both leukaemia and breast cancer models^110^. Glucose competition within the TME also represents a major metabolic barrier. Engineering CAR T cells to overexpress the high-affinity glucose transporter GLUT3 enables efficient glucose uptake under nutrient-limited conditions, thereby sustaining glycolytic metabolism and enhancing tumour-killing activity^32^. Lipid metabolism also plays a central role in CAR T cell persistence. Reprogramming the TAGLN2–FABP5 axis enhances fatty acid uptake and mitochondrial respiration in T cells^111^. Conversely, ER stress within the TME suppresses TAGLN2 expression and contributes to T cell dysfunction^111^. CAR T cells engineered to maintain high TAGLN2 expression demonstrate improved metabolic fitness and therapeutic efficacy in ovarian cancer models^111^. Meanwhile, brain tumours are characterized by a highly hypoxic microenvironment compared with subcutaneous tumours, which constrains oxidative phosphorylation and limits mitochondrial ATP-generating capacity in infiltrating T cells^112^. To metabolically reprogram CAR T cells to better withstand these conditions, the AMPK activator metformin and the mTOR inhibitor rapamycin were combined during the CAR T cell expansion phase^112^. This metabolic preconditioning promotes activation of the AMPK–PGC-1α axis, driving mitochondrial biogenesis and increasing spare respiratory capacity, thereby enhancing metabolic flexibility under nutrient- and oxygen-restricted conditions^112^. As a result, CAR T cells display improved bioenergetic fitness and elevated IFNγ production^112^. Moreover, these metabolically optimized CAR T cells reduce the accumulation of monocytic myeloid-derived suppressor cells within the TME, ultimately prolonging survival in a glioblastoma multiforme mouse model^112^. Notably, similar metabolic remodelling was observed in human-derived CAR T cells, highlighting the translational potential of this strategy^112^.

Recent studies suggest that type 2 cytokine signalling, particularly through IL-4, can be harnessed to enhance the survival and metabolic fitness of CAR T cells within the TME. Administration of a long-acting IL-4 fusion protein (Fc-IL-4) together with CAR T cells activates STAT6 and mTOR signalling pathways, resulting in enhanced glucose metabolism and increased intracellular NAD^+^ levels in CD8^+^ T cells through an LDHA-dependent mechanism^102^. These metabolic adaptations restore T cell functionality and improve anti-tumour responses. Another strategy exploits the immunosuppressive cytokine environment of solid tumours by engineering CAR T cells with inverted cytokine receptors. In this design, the extracellular domain of the IL-4 receptor is fused to the intracellular signalling domain of the IL-7 receptor (4/7 inverted cytokine receptor)^113^. As a result, tumour-derived IL-4 — normally an inhibitory signal — triggers IL-7-like survival and proliferative signalling within CAR T cells^113^. MUC1-targeted CAR T cells expressing this receptor exhibit enhanced proliferation and sustained anti-tumour activity in breast cancer models despite the presence of high IL-4 levels in the TME^113^. Consistent with these observations, single-cell multi-omics analyses of CAR T cell therapy in patients with acute lymphoblastic leukaemia reveal that type 2 immune signalling contributes to long-term CAR T cell persistence^114^. In addition to the established role of type 1 immunity, IL-4-associated signalling pathways help to maintain population homeostasis and support the expansion of functionally competent T cells. CAR T cells exposed to IL-4 display reduced functional exhaustion and improved cellular fitness, suggesting that controlled activation of type 2 cytokine pathways may represent an additional strategy for enhancing CAR T cell durability.

Together, these findings indicate that overcoming CAR T cell exhaustion in solid tumours requires coordinated engineering strategies that reshape transcriptional, epigenetic, metabolic and cytokine signalling programs. CAR T cells pre-conditioned along these axes during in vitro manufacturing are likely to exhibit greater synergy when combined with checkpoint blockade. From this perspective, the combination of in vitro reprogramming and immune-checkpoint inhibition represents a promising approach to enhancing the efficacy of CAR T cell therapy within the TME.

## Conclusion and future directions

Although substantial progress has been made in understanding T cell exhaustion, important conceptual and technical challenges remain. A major limitation arises from the fact that exhaustion represents a continuous differentiation program rather than a discrete cellular state. Nevertheless, exhausted T cells are still defined across laboratories using heterogeneous combinations of inhibitory receptors or functional readouts, which complicates direct comparison between studies and hinders the development of unified mechanistic models.

Future studies should conceptualize T cell exhaustion within an integrated framework that incorporates cellular states, clonal lineages and spatial context within TME. This perspective recognizes that exhaustion emerges from dynamic interactions between differentiation trajectories, clonal lineage relationships and the spatial organization of immune niches within tumours. Addressing these questions will require systematic integration of multi-omics technologies, including single-cell transcriptomics, TCR repertoire profiling and chromatin accessibility mapping (multiome approaches combining scRNA-seq, TCR-seq and ATAC-seq), together with spatial transcriptomics and in situ phenotyping platforms. These approaches enable direct linkage of transcriptional and epigenetic states to clonal lineage and precise intratumoural localization, revealing how distinct exhausted T cell (T_EX_) states arise and evolve within tumour parenchyma, TLS, tumour-draining lymph nodes, stromal compartments and myeloid-rich niches. In parallel, humanized tumour models and barcode-based lineage-tracing strategies will be essential for temporally resolving differentiation trajectories and transition relationships among progenitor, intermediate and T_PEX_ cells.

Another major priority for the field is the establishment of minimal reporting standards to harmonize the definition of exhaustion. At a minimum, future studies should report evidence of sustained antigen exposure, TCR clonality and clonal expansion, combinatorial expression of inhibitory receptors rather than single markers, hierarchical loss of effector functions, inferred differentiation trajectories, exhaustion-associated epigenetic states and spatial context within the TME. Adoption of such shared criteria would facilitate transition of exhausted T cells research from descriptive phenotyping towards mechanism-driven frameworks and allow more meaningful comparison across tumour types and experimental systems.

Together, this review highlights that T cell exhaustion represents a multidimensional process shaped by convergent signalling pathways and the spatially organized TME. Precise dissection of these complex programs will require the integration of multi-omics, spatial profiling, and lineage-tracing technologies capable of simultaneously resolving cellular states, clonal histories and tissue niches. We therefore propose the establishment of standardized frameworks to decode exhaustion across states and spatial contexts. Such approaches will enable the rational design of next-generation immunotherapies that simultaneously account for T cell differentiation states and the TME, ultimately paving the way toward more durable and personalized cancer immunotherapy strategies.
